# Draft Genome Sequence of the Multidrug-Resistant Clinical Isolate *Dermabacter hominis* 1368

**DOI:** 10.1128/genomeA.00728-14

**Published:** 2014-07-24

**Authors:** Andreas Albersmeier, Christina Bomholt, Alina Glaub, Christian Rückert, Francisco Soriano, Isabel Fernández-Natal, Andreas Tauch

**Affiliations:** a Institut für Genomforschung und Systembiologie, Centrum für Biotechnologie (CeBiTec), Universität Bielefeld, Bielefeld, Germany; b Public Health School of Physiotherapy, Autonomous University of Madrid, Madrid, Spain; c Department of Clinical Microbiology, Complejo Asistencial Universitario de León, Sacyl León, Spain; d Institute of Biomedicine (IBIOMED), University of León, León, Spain

## Abstract

*Dermabacter hominis* is a common colonizer of the healthy human skin and is rarely detected as an opportunistic human pathogen. The genome sequence of the multidrug-resistant *D. hominis* strain 1368, isolated from blood cultures of a pyelonephritis patient, provides insights into the repertoire of antibiotic resistance genes.

## GENOME ANNOUNCEMENT

The species *Dermabacter hominis* was established by Jones and Collins in 1988 to classify coryneform bacteria from healthy human skin on the basis of their biochemical characteristics (1). Subsequent reports assigned coryneform bacteria of the Centers for Disease Control and Prevention (CDC) group 3 and group 5 to the new species *D. hominis*, indicating the potential clinical significance of this microorganism (2, 3). CDC group 3 and group 5 coryneforms were primarily isolated from human blood cultures and less commonly from other body sites (2, 3). Additional reports documented that *D. hominis* was recovered from a brain biopsy specimen (4), the peritoneal fluid of a dialysis patient (5), blood cultures of two bacteremic patients (6), recurrent cutaneous abscesses (7), human semen specimens (8), and cases of fatal septicemia (9) and chronic osteomyelitis (10).

A recent study reviews clinical features of *D. hominis* infections and contains data on 14 patients attending a tertiary hospital (11). *D. hominis* was mostly isolated from blood cultures and peritoneal dialysis catheter exit sites. *In vitro* assays demonstrated that the isolates were susceptible to linezolid, rifampin, and vancomycin, whereas their resistance profile to other antibiotics was variable. With the exception of one isolate, all *D. hominis* strains were resistant to the lipopetide antibiotic daptomycin. Isolate no. 5 presented multiple resistances to antibiotics, including daptomycin (11). To gain access to the repertoire of antibiotic resistance genes of isolate no. 5, we sequenced the genome of this multidrug-resistant strain that was renamed as *D. hominis* 1368.

*D. hominis* 1368 was isolated from blood cultures of a 29-year-old patient suffering from lymphoma and pyelonephritis (11). The bacterium was grown in brain-heart infusion broth at 37°C to purify genomic DNA with the Genomic DNA buffer set and the Genomic-tip 500/G system (Qiagen). A DNA sequencing library was constructed on the basis of the Nextera DNA sample preparation kit (Illumina) and was sequenced by the 2×250 nucleotide paired-end approach using the MiSeq reagent kit v2 and the MiSeq desktop sequencer (Illumina). This whole-genome shotgun sequencing resulted in 2,163,261 reads and 504,763,054 detected bases. The reads were preprocessed by quality-trimming in such a way that the last five nucleotides at the 3′ end had a Phred quality value of ≥30 (12). The quality-trimmed reads were assembled with the GS De Novo Assembler software (version 2.8) to yield 56 contigs.

The current draft genome sequence of *D. hominis* 1368 has a size of 2,507,630 bp. The annotation of this nucleotide sequence was performed with the NCBI Prokaryotic Genome Annotation Pipeline and the GeneMarkS+ software (version 2.3), revealing 2,227 protein-coding genes, 21 pseudogenes, 1 non-coding RNA gene, and 48 tRNA genes. Strain 1368 contains typical corynebacterial antibiotic resistance determinants (11, 13), indicating that horizontal gene transfer between *D. hominis* and corynebacteria of the human skin flora plays an important role in the development of multidrug resistance. The genome sequence of *D. hominis* 1368 therefore helps to further the understanding of the variability of resistances to clinically relevant antimicrobials in this species (14–16).

### Nucleotide sequence accession numbers.

This whole-genome shotgun project has been deposited in GenBank under the accession no. JDRS00000000. The version described in this genome announcement is the first version, JDRS01000000.1.
